# Molecular Mechanisms of Environmental Metal Neurotoxicity: A Focus on the Interactions of Metals with Synapse Structure and Function

**DOI:** 10.3390/toxics9090198

**Published:** 2021-08-27

**Authors:** Asuncion Carmona, Stéphane Roudeau, Richard Ortega

**Affiliations:** University of Bordeaux, CNRS, CENBG, UMR-5797, F-33170 Gradignan, France; acarmona@cenbg.in2p3.fr (A.C.); roudeau@cenbg.in2p3.fr (S.R.)

**Keywords:** neurotoxicity, synapse, metal, arsenic, cadmium, lead, manganese, mercury

## Abstract

Environmental exposure to neurotoxic metals and metalloids such as arsenic, cadmium, lead, mercury, or manganese is a global health concern affecting millions of people worldwide. Depending on the period of exposure over a lifetime, environmental metals can alter neurodevelopment, neurobehavior, and cognition and cause neurodegeneration. There is increasing evidence linking environmental exposure to metal contaminants to the etiology of neurological diseases in early life (e.g., autism spectrum disorder) or late life (e.g., Alzheimer’s disease). The known main molecular mechanisms of metal-induced toxicity in cells are the generation of reactive oxygen species, the interaction with sulfhydryl chemical groups in proteins (e.g., cysteine), and the competition of toxic metals with binding sites of essential metals (e.g., Fe, Cu, Zn). In neurons, these molecular interactions can alter the functions of neurotransmitter receptors, the cytoskeleton and scaffolding synaptic proteins, thereby disrupting synaptic structure and function. Loss of synaptic connectivity may precede more drastic alterations such as neurodegeneration. In this article, we will review the molecular mechanisms of metal-induced synaptic neurotoxicity.

## 1. Introduction

Three metals, lead (Pb), mercury (Hg), and cadmium (Cd), and the metalloid arsenic (As), have been listed among the ten chemicals of major public health concern by the World Health Organization (WHO) [1]. As, Pb, Hg, and Cd are ranked as first, second, third, and seventh, based on their frequency, toxicity, and potential for human exposure in the priority list of the USA Agency for Toxic Substances and Disease Registry [2]. They all cause neurotoxic effects [3]. Manganese (Mn) is another neurotoxic element of growing concern in terms of environmental overexposures [4]. Sources of human environmental exposure are diverse, from air, food, or drinking water. Environmental exposure to neurotoxic metals is a global health problem affecting millions of people worldwide. For instance, at least 140 million people in 50 countries have been drinking water containing As at levels above the WHO guideline [5]. Depending on the period of exposure over a lifetime, environmental metals affect neurodevelopment [6,7], neurobehavior [8], cognition [9], or are involved in the etiology of neurodegenerative diseases such as Alzheimer’s (AD) and Parkinson’s (PD) diseases [10,11,12,13,14,15,16]. In particular, prenatal and early childhood exposure to environmental metals such as As, Pb, Mn, Cd, or Hg has emerged as strong candidate etiological factors in autism spectrum disorder (ASD) [6,17]. Increased fetal and postnatal uptake of Pb are observed in ASD cases [18]. In adults, high blood levels of As, Hg, and Cd are associated with increased risk of AD [19,20].

The known key molecular pathways of metal-induced toxicity in cells, including neurons, involve the production of reactive oxygen species (ROS), the interaction with sulfhydryl chemical groups (-SH) in proteins, and the competition with binding sites of essential metals (e.g., Fe, Cu, Zn) [21]. Whatever the route of exposure, As, Pb, Hg, and Mn can pass the blood–brain barrier (BBB) and reach the central nervous system (CNS) [11,22]. Cd can barely pass the BBB in adults; however, the BBB is not fully functional in the developing brain [23]. In adults, Cd can be uptaken in the CNS directly through the olfactory pathway and can also alter the BBB contributing to the pathogenesis of neurodegenerative diseases [24]. In neurons, metal-induced molecular damages will affect specific neuronal functions through, for instance, interactions with synaptic vesicles, ion channels, metabolism of neurotransmitters, intracellular signaling pathways, neurotransmitter receptors, transcription machinery regulating synaptic plasticity, or through a combination of these mechanisms [25,26].

In particular, neurotoxic effects will result in altered synaptic transmission and synaptic plasticity. These mechanisms of metal-induced synaptic neurotoxicity are receiving increasing attention. Such mechanisms are consistent with new paradigms on the etiology of major neurological disorders such as ASD, schizophrenia, or AD, suggesting that synaptic alterations may occur early in the pathological process [27], and could involve impairments of the cytoskeletal and mechanical integrity of synaptic structures [28,29,30]. This review article will focus on the molecular mechanisms of interaction of environmental toxic metals with the synaptic structure and function. We will review the state-of-the-art knowledge on metal interactions with neurotransmitter receptors and with cytoskeletal and scaffolding synaptic proteins.

## 2. Metal Interactions with Neurotransmitter Receptors

Environmental neurotoxic metals can impair neurotransmitter receptors functions as reviewed in two important articles in this field of research [25,26]. Neurotoxic metals can interact with neurotransmitter receptors by a variety of mechanisms including the modification of their gene and/or protein expression, the indirect disruption of their functions following ROS production, or the direct competition with physiological ions binding sites on the proteins. Here, we will summarize the research works studying molecular interactions between As, Cd, Pb, Mn, and Hg with neurotransmitter receptors, such as the ionotropic glutamate receptors (N-methyl-D-aspartate (NMDA), α-amino-3-hydroxy-5-methyl-4-isoxazolepropionic acid (AMPA), and kainate receptors), the γ-aminobutyric acid (GABA) receptors and the dopamine (DA) receptors.

### 2.1. Arsenic

**Figure 1:**
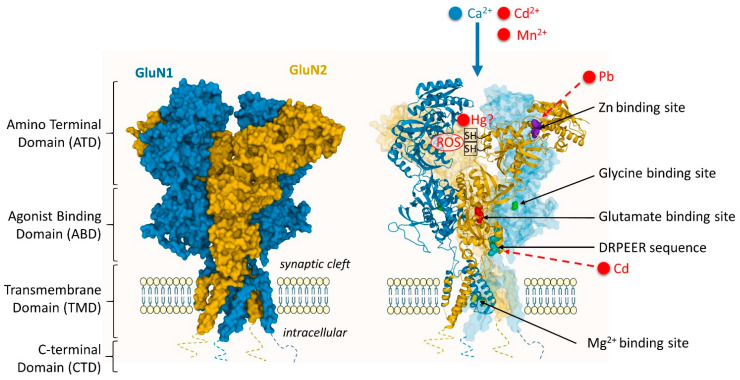
Interaction sites of environmental metal toxicants with the NMDA receptor. Cd^2+^ neuronal uptake is mediated by the NMDAR following its stimulation. Similarly, Mn^2+^ can permeate the plasma membrane through the NMDAR. Cd can bind directly to the DRPEER sequence in the extracellular domain (in the ABD/TMD linker) of the GluN1 subunit and inhibit NMDA-mediated current. Pb competes with zinc to the zinc-binding site of the GluN2 subunit and alters the receptor function. In the case of Hg, there are some indications for interactions with the cysteine -SH groups involved in the control of NMDAR activity, although this suggestion still needs experimental evidence. In most cases, metals could induce ROS that would interact with the –SH groups of the NMDAR. Structure of the GluN1/2B NMDAR (Protein Data Bank accession 4PE5 [32]). Figure inspired from Hansen et al. [52].

Changes in glutamatergic, cholinergic, and dopaminergic systems have been linked to As exposure, resulting in impaired synaptic transmission, as recently reviewed [22]. Arsenic exposure alters the expression of the NMDA receptor (NMDAR) in the hippocampus of animal models, as evidenced in several studies. The NMDAR is a heterotetramer typically composed of GluN1 subunits with GluN2 subunits, or of GluN1 and a mixture of GluN2/GluN3 subunits (Figure 1) [31,32]. Subunit composition of the NMDAR varies during development [31]. Arsenic induces complex modifications in NMDAR subunit expression, with effects of up- or down-regulation at the receptor subunit level, depending on the period of As-exposure during neurodevelopment. For instance, the expression of several NMDAR subunits is downregulated at the protein level following As exposure, as evidenced for GluN1 [33,34], GluN2A [34,35,36], and GluN2B subunits [34,36,37]. Changes are, however, complex, with concomitant up- and down-regulations following As-exposure, e.g., GluN1 can be down-regulated while GluN2A is up-regulated [33], GluN2B down-regulated while GluN2A is up-regulated [37], or GluN2A down-regulated while GluN1 remains unchanged [35]. This complex picture of As effects on NMDAR subunits expression in function of neurodevelopment stages is exemplified by the comparison of expression at different postnatal periods. On mice exposed to As through drinking water, Western blotting reveals a downregulation of GluN2A 15 days after birth and an upregulation after 90 days, while GluN2B is down-regulated 15 days after birth but no significant changes are observed after 90 days [36].

A similar effect is observed for the AMPA receptor (AMPAR), As-exposure leading to the down-regulation of a specific subunit of the AMPAR [38], such as GluA1, as observed both at the mRNA and protein levels [34,39]. The inhibition of glutamate receptors expression alters synaptic plasticity such as long-term potentiation (LTP), learning, and memory, and is associated with an increase in extracellular glutamate levels [36,38].

Although less investigated than glutamate receptors, changes in the expression of other neurotransmitter receptors following As exposure are also reported. Inorganic As decreases the mRNA expression levels of α7 nicotinic receptors in rats [40], and alters the development of the cholinergic and dopaminergic systems [41]. Chronic exposure to As at a high dose in mice decreases mRNA expression of DA-D2 receptors although there are no changes in DA-D1 receptors in striatum [42].

### 2.2. Cadmium

Cd exposure affects glutamate, acetylcholine, GABA, and DA neurotransmitter receptors functions in the brain. Cd uptake in neurons is mediated by the NMDAR voltage-dependent calcium channels as indicated by increased Cd influx following stimulation with glutamate or NMDA and glycine [43] (Figure 1). It is also known that extracellular Cd binds directly to the DRPEER sequence of the NMDA channel and inhibits currents in a concentration-dependent manner [44] (Figure 1). The DRPEER sequence is a cluster of charged residues and a proline located extracellularly. This sequence is unique to the GluN1 subunit of the NMDAR and responsible for the high Ca^2+^ flux rate [45]. In addition, Cd impairs the AMPAR-mediated synaptic transmission and short-term plasticity in the rat hippocampus [46].

Cd also interacts with muscarinic acetylcholine receptors. Cd causes cell death in primary cholinergic neurons from the basal forebrain by silencing the muscarinic receptor M1 [47]. In a following study from the same authors, it was shown that Cd effects on muscarinic receptors disruption were induced by oxidative stress [48].

Early studies evidenced that Cd inhibits the neuronal GABAA receptor channel complex through a binding site that was distinct from the recognition sites for GABA and for pharmacological agents [49]. On the other hand, Cd exposure modifies the expression of GABAA receptors in animal studies [50]. In particular, different protein expression levels of GABAARα5 and GABAARδ were observed in the hippocampus of mice offspring after Cd exposure during pregnancy and lactation suggesting that GABAARα5 is more sensitive to the environmental pollutants at puberty and young adulthood, whereas GABAARδ may reflect the accumulation of the environmental pollutants at adulthood.

Finally, Cd neurotoxicity induces motor dysfunctions that have been related to Cd selective effects on DA receptors. Cd exposure decreases mRNA and protein expression of DA-D2 receptors in the striatum of rat brains, whereas levels of expression for DA-D1 receptors were unchanged [51]. Moreover, molecular docking experiments revealed that Cd could directly bind at the competitive site of dopamine on DA-D2 receptors. This selective inhibition of DA-D2 receptors is similar to the one described for As exposure [42].

### 2.3. Lead

There is a large body of evidence for the interaction of Pb with glutamatergic signaling affecting more the hippocampus than other brain regions [25]. The adverse effects of Pb-exposure on glutamate neurotransmission have long been identified and involve a reversible inhibition of the NMDA-activated calcium channel current [53,54]. Pb-exposure could block the NMDAR, preventing the influx of Ca^2+^ into the postsynaptic neuron, by interaction with zinc neurophysiology [55] (Figure 1). Zinc is a physiological allosteric inhibitor of NMDAR function [56]. The presence of Pb decreases the inhibitory effect of Zn, suggesting that the two metals compete for binding in the zinc-binding site of the NMDAR [57]. Since Zn binds with high affinity at a regulatory site on the GluN2A subunit, but with lower affinity to the GluN2B subunit, this suggests a preferential sensitivity of GluN2A NMDAR for Pb [57,58,59].

Pb exposure may also affect the expression of NMDAR. Pb exposure induces a decreased expression of the NMDAR subunit GluN2A in synapses from hippocampal neurons, and an increased targeting of subunit GluN2B to dendritic spines [60]. This effect is particularly critical during neuronal development. Chronic developmental Pb exposure results in decreased levels of GluN2A mRNA and altered levels of GluN1 mRNA in the hippocampus [61,62,63] and altered expression of GluN1 splice variants [64]. During brain development, there is a shift of GluN2B- to GluN2A-containing NMDAR. These data suggest that Pb delays the normal developmental switch of increased GluN2A incorporation in NMDA receptors during synapse maturation [59].

Pb may prevent the expression of the NMDAR gene by substituting for zinc in the specificity protein-1 (Sp1), a ubiquitously expressed zinc-finger transcription factor which upregulates NMDAR transcription. Pb competition for binding in the zinc finger region of Sp1 could induce conformational changes that may alter the expression of NMDAR [55]. This hypothesis is based on the observation that Pb exposure alters the binding of Sp1 to DNA, that Zn supplementation has a protective effect and that GluN1 expression globally follows Sp1-to-DNA binding [65].

Electrophysiological studies showed that LTP induction was affected in Pb exposed rats, and this was probably due to excitatory synaptic transmission impairment [66]. This study has evidenced that NMDA and AMPA receptor-mediated current was inhibited, and GluN2A and phosphorylated GluA1 expression were decreased. Moreover, morphological changes were observed with a decline in dendritic spines density and in spine maturation in Pb exposed rats.

The action of Pb on glutamate release, NMDAR function, and structural plasticity can underlie perturbations in synaptic plasticity significantly affecting LTP, and contributing to impairments in hippocampus-mediated learning and memory functions [59,67,68,69].

NMDA receptors are more sensitive to Pb inhibition than other glutamate channels but Ca^2+^-permeable AMPAR may also be involved since Pb exposure reduces the expression of the glutamate receptor subunit GluA2 [70]. This result was further confirmed showing that the expression of GluA1, GluA2, GluA3, and GluA4 AMPAR subunits is decreased by Pb exposure in cortical neurons, but with a more pronounced decrease in GluA2 expression [71]. Other results showed that GluA1 and GluA2 expressions are increased in tetrodotoxin-Pb induced synaptic scaling [72].

Although Pb affects mainly the glutamate receptors, the consequences of Pb exposure on DA or GABA systems have also been studied [25]. In the developing rat brain, Pb-exposure can modify the aminergic system, most likely as result of decreased monoamine oxidase (MAO) activity [73]. MAOs are important enzymes in the breakdown of monoamines and in the regulation of monoamine neurotransmission. The expression of MOAs is sensitive to the exposure of various metals, such as Pb [73], but also Mn [74], Hg [13], and uranium [75]. Metal-induced MAO inhibition can thus indirectly impact the aminergic pathways and the expression of dopamine receptors. It has been hypothesized that Pb substitution for Zn may directly target the DA-D1- and -D2 receptors known to contain Zn-binding sites [55]. However, the direct substitution of Zn by Pb in DA receptors still needs to be demonstrated experimentally.

Rats exposed to low levels of Pb have reduced Ca-dependent GABA release in the hippocampus [76]. Similarly, Pb inhibits action potential-dependent GABA release in rat hippocampal slices, possibly through the involvement of voltage-gated calcium channels [77]. However, in other studies of Pb exposure, the GABA synaptic transmission was normal [66].

### 2.4. Manganese

Mn can interfere with the dopaminergic, cholinergic, glutamatergic, and GABAergic systems [78,79,80]. Mn disturbs these neurotransmitter systems through multiple mechanisms including the direct interaction with neurotransmitter receptors. Mn is a NMDA calcium channel blocker as evidenced in cultured neurons [81], and in different brain regions (cerebral cortex, hippocampus, striatum, and cerebellum) on Mn exposed rats [82]. On the other hand, Mn inhibits the mRNA and protein expression of NMDAR GluN1, GluN2A, and GluN2B subunits in primary cultured neurons, with a larger decrease of the GluN2A subunit compared to the two others [83]. In vivo exposure to Mn decreased the mRNA and protein expression of GluN1 and GluN2A subunits in the striatum of mice [84], and also decreased the protein levels and mRNA expression of NMDAR GluN1, GluN2A, and GluN2B in the rat hippocampus [85].

However, studies in animal models of Mn-induced brain pathology have shown that the glutamatergic system appear to be mostly unaffected by chronic Mn exposure [86,87]. Although the glutamatergic receptors might not be the primary target of Mn neurotoxicity, they might play a role in Mn accumulation in the central nervous system. Mn can permeate the plasma membrane through the NMDAR [72] (Figure 1). The entry of Mn into the brain is accelerated by the activation of NMDAR (but not AMPAR) in glutamatergic neurons [88]. Another mechanism of Mn neurotoxicity involving glutamatergic receptors is related to Mn induced alpha-synuclein overexpression, which results in the phosphorylation and downregulation of the GluN2B subunit, and the consequent impaired NMDAR signaling [89].

Interactions of Mn with GABA receptors have also been investigated. Mn exposure decreases GABAA receptor subunit protein expression in the hypothalamus from immature female rats [90]. In vitro experiments on Mn exposed neurons revealed a suppression of GABAA receptors and induction of GABAB receptors, leading to the accumulation of alpha-synuclein [91]. However, similarly to the glutamatergic system, the GABAergic system might not represent the main target of Mn neurotoxicity and more pronounced neurotoxicity is observed towards the dopaminergic system [59,86,92].

Mn effects on DA receptors have been investigated in a variety of animal models. In Mn-treated mice, the mRNA and protein levels expression of the DA-D2 receptor is increased in the striatum, in a dose dependent way, while the expression of DA-D3 or DA-D4 receptors remains unchanged [93]. In a rat experimental model of early life Mn exposure, decreased expression of DA-D1 receptors in nucleus accumbens and dorsal striatum, as well as increased expression of DA-D2 receptors in the prefrontal cortex, were associated with behavioral and learning deficits [94]. In rats exposed to Mn throughout the postnatal days, a persistent increase of DA-D2 receptors protein expression in the dorsal striatum is found [95]. The authors suggest that early Mn exposure depresses presynaptic dopaminergic function, reduces DA levels, causing an up-regulation of DA-D2 receptors expression and a dysregulation of DA-associated signaling pathways. In the striatum of mice exposed to Mn, immunohistochemical activities, protein levels, and mRNA expression of DA-D1 receptors are decreased [84]. The interactions between DA-D1 and NMDAR are inhibited in this animal model resulting in learning and memory dysfunction via injury of striatum. Overall, these studies suggest that Mn exposure induces a decrease of the DA-D1 receptors expression in the frontal cortex as well as an increase of DA-D2 receptors expression in the striatum.

In Mn exposed welders and workers, a PET (positron emission tomography) molecular imaging study of basal ganglia DA-D2 receptors revealed an increased binding of the DA-D2 receptor specific antagonist [11C](N-methyl)benperidol in the substantia nigra, compared to non-exposed workers [96]. This study confirms in humans the link observed in animal models between Mn exposure and DA-D2 receptor increased expression.

### 2.5. Mercury

Hg interacts with a wide range of neuronal targets including NMDA, GABA, DA, and acetylcholine (ACh) receptors. For example, methyl-Hg (MeHg) induced neuronal toxicity is thought to involve glutamate-mediated excitotoxicity. Activation of NMDAR following MeHg exposure has been reported in developing cortical neurons [97], and in the frontal cortex of adult rats [98]. As indicated in rats, overstimulation of NMDAR following Hg exposure may also contribute to neurotoxicity by inducing excessive dopamine release in the striatum [99]. In hippocampal neurons, HgCl_2_ and thimerosal (an organomercurial compound used as vaccine preservative) reduce NMDA-evoked currents, with higher toxicity for HgCl_2_ [100]. It was proposed that Hg could interact with the cysteine -SH groups, crucial for regulating NMDAR function, rendering the NMDAR dysfunctional (Figure 1). This postulate, however, still needs to be confirmed experimentally.

In Purkinje and granule cells of rat cerebellar slices, MeHg alters GABAA receptor-mediated inhibitory synaptic transmission at both presynaptic and postsynaptic sites [101]. In cultured hippocampal neurons, HgCl_2_ markedly and rapidly potentiates the GABAergic currents while thimerosal works slowly and reduces GABA responses [100]. It was suggested that mercurial compounds most likely interact with cysteine -SH residues at the GABAA receptor complex, critical for its gating properties.

Hg compounds can also alter the brain dopaminergic system. Thimerosal exposure induces a decrease in the density of striatal DA-D2 receptors in exposed rats [102]. In rats exposed to MeHg there is a decrease of DA-D1 receptors in both cortex and striatum, and an increase of DA-D2 receptors in cortex [103]. Here again, the presence of –SH groups in the DA receptors might be the target of Hg binding as shown in biochemical assays exhibiting very stable and non-reversible binding of Hg to the DA-D2 receptor [104].

There are also evidence from animal studies for Hg to affect the global population of ACh receptors. Hg inhibits muscarinic cholinergic ligand binding to the mACh receptor [105], and preferentially affects ACh receptor subtypes M1 and M2 levels in the occipital cortex [106].

## 3. Metal-Interactions with Proteins of the Synaptic Structure

**Figure 2:**
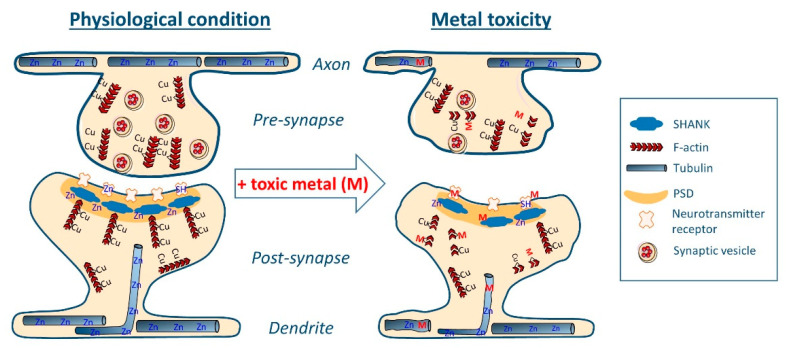
Schematic representation of synaptic toxicity mechanisms by direct competition of toxic metals (M) with physiological metal binding sites (Cu, Zn) within cytoskeletal proteins (e.g., tubulin, F-actin and/or F-actin binding proteins), PSD scaffold proteins (e.g., SHANK), and neurotransmitter receptors (e.g., NMDAR). These metal–protein interactions can damage the synaptic structure and lead to loss of connectivity between neurons.

The disorganization of the synaptic cytoskeletal structure is involved in numerous neurological dysfunctions, including neurodevelopmental disorders [107,108,109], and neurodegenerative diseases such as AD [110,111]. Recent findings from our team suggest that Cu and Zn physiologically interact with dendro-synaptic tubulin and F-actin to control synapse formation and stability [112,113]. On the other hand, the Shank family of proteins, which are important scaffold proteins involved in the post-synaptic density (PSD) structure, requires Zn to regulate their assembly [114]. As a corollary to these physiological functions, toxic metals could bind directly to cytoskeleton and scaffold proteins (actin, tubulin, SHANK3), possibly by competing with Cu- and/or Zn- binding sites, resulting in the structural disorganization of synapses, thus leading to impairments in synaptic connectivity (Figure 2).

Actin and tubulin are the most widely expressed cytoskeleton proteins and are involved in the formation, plasticity and stability of synapses [115,116]. Dynamic polymerization or depolymerization of actin filaments (F-actin) serve as driving force for the formation or retraction of dendritic spines. Microtubules are formed by the polymerization of αβ-tubulin heterodimers and are located mainly in axons and dendrites but also in synapses (Figure 2).

On the other hand, SHANK3 protein is a major scaffold protein involved in the multi-protein complex assembly of the PSD. Deletions, mutations, or downregulation of SHANK3 gene are linked to ASD [117]. Zn binds to SHANK3 allowing the PSD assembly [114], and promoting synaptogenesis [118]. Zn deficiency dysregulates the synaptic SHANK3 assembly and may contribute to ASD [119], whereas Zn supplementation could restore neuronal functions [120,121]. Reduced fetal and postnatal uptake of Zn and increased uptake of Pb are observed in ASD cases [18]. In vitro, the exposure to a biometal profile characteristic for ASD (low Zn and high Cd, Pb and Hg) resulted in the reduction of SHANK genes expression along with a reduction of synapse density [122].

### 3.1. Arsenic

The administration of sodium meta-arsenite to Wistar rats results in the destabilization of the cytoskeletal framework observed in sciatic nerves [123]. Protein analysis of the sciatic nerves revealed changes in neurofilament-low (NF-L) protein expression together with the hyper-phosphorylation of NF-L and microtubule-associated protein TAU proteins. Sodium arsenite suppresses neurite outgrowth in mouse neuroblastoma cells, a mechanism induced by the decreased mRNA levels of TAU and tubulin (TUBB5) cytoskeletal genes [124]. In NT2 postmitotic neurons, sodium arsenite exposure significantly reduces neuronal differentiation and the expression of β-tubulin type III protein [125]. These results point out tubulin as a target of As neurotoxicity. Information on molecular mechanisms of toxicity are available from studies of non-neuronal systems. For example, in cultured lung fibroblast, sodium arsenite induces a dose-dependent disassembly of microtubules by targeting the tubulin sulfhydryl groups [126].

### 3.2. Cadmium

CdCl_2_ induces the disassembly of the cytoskeleton in a variety of cultured neuronal cells, targeting both the actin [127], and the microtubules [128] networks. Cd induces the destruction of microtubules and decreases acetylated tubulin levels in primary rat cortical neurons [129]. Cd down-regulates the gene expression of microtubules dynamics and microtubules motor-based proteins in a neuronal human cellular model [130]. Again, information on molecular interactions with cytoskeleton proteins is available from studies of non-neuronal systems. In cultured lung cells, Cd at concentrations similar to environmental exposures (0.5–1 µM), caused significant oxidation of peptidyl cysteines of proteins regulating actin cytoskeleton and increased filamentous actin polymerization [131].

### 3.3. Lead

Exposure to inorganic Pb reduces the expression of tubulin and induces the disorganization of the cytoskeleton in primary neuronal cells and neonatal brain [132]. Pb toxicity is generally driven by its binding to sulfhydryl proteins, as shown in non-neuronal cells [21]. In porcine brain and in cultured fibroblasts, triethyl lead chloride inhibits microtubule assembly and depolymerizes preformed microtubules by interaction with thiol groups of the tubulin dimer [133]. Moreover, morphological studies showed that density of dendritic spines declined by about 20% in Pb-treated rats, the remaining spines showing an immature form [66].

### 3.4. Manganese

Mn exposure induces dramatic changes in the neuronal cytoskeleton, even at sub-cytotoxic concentrations, in dopaminergic and GABAergic neurons [134]. Environmentally relevant concentrations of Mn impairs cytoskeletal morphology and structure in adult neuronal stem cells by decreasing F-actin polymerization [135]. The molecular mechanisms underlying these effects are not known.

### 3.5. Mercury

In neuroblastoma cells, Hg induces microtubule disruption at concentrations markedly lower than in fibroblasts, indicating a particular sensitivity of nerve cells to Hg [136]. Inorganic Hg exposure results in reduced tubulin expression in primary neuronal cells [124]. A similar reduction in tubulin expression is observed on NT2 postmitotic neurons after MeHg exposure [125]. Using immobilized metal affinity chromatography, thirty-eight Hg-binding proteins have been identified in human neuroblastoma cells, among them tubulin (both α and β subtypes), and various isoforms of actin [137]. In fish muscle, β-actin is one of the main Hg-binding protein together with tropomyosin, a protein also required in the formation of actin filaments [138]. Tubulin sulfhydryl groups have been proposed as the target of Hg toxicity [139]. In vitro exposure of primary cultures of rats to MeHg resulted in neuronal network fragmentation and microtubule depolymerization detected as early as within 1.5 h of MeHg exposure, long before the occurrence of nuclear condensation (6–9 h) [140]. In cortical neurons, Hg exposure compromises the cytoskeleton components, mainly the β-tubulin [141].

More generally, the mechanism of metal induced damage to cytoskeleton proteins may apply not only to neurons but also to other brain cells such as astrocytes. For example, Hg and Mn exposure result in cell swelling in cultured astrocytes, a morphological change also found in AD [142,143]. Mn, Cd, or Hg exposure can alter the cytoskeletal structure of neural stem cells during their differentiation into astrocytes [129,144]. Since astrocytes play an active role in the synaptic organization as conceptualized in the model of the tripartite synapse [145], metal-induced disorganization of the astrocytic cytoskeleton could participate to the impairment of synaptic functions.

## 4. Conclusions

Neurotoxic environmental metals could directly alter synaptic structure by disrupting the cytoskeleton organization (e.g., F-actin, microtubules), or scaffolding proteins in the postsynaptic compartment (e.g., SHANK3), or indirectly by modifying the levels of expression of neurotransmitters receptors (e.g., glutamate, DA, GABA and ACh receptors) themselves involved in the regulation of synaptic plasticity.

These structural impairments could be due to metal-induced production of ROS, generally targeting sulfhydryl (-SH) groups of proteins involved in synaptic structure. Sulfhydryl groups in neurotransmitter receptors and in cytoskeleton proteins are also potential sites of direct metal interactions. In addition, competition of toxic metal with essential metal (Cu, Zn) binding-sites in synaptic proteins (e.g., NMDAR, tubulin, SHANK3) might also be important molecular targets of metals neurotoxicity.

Overall, these effects would cause the disorganization of the synaptic structure, resulting in synaptic impairments, loss of synaptic connectivity and neurological dysfunctions. This mechanism may apply to a wide variety of neurological disorders, including neurodegenerative diseases such as AD, or developmental disorders (e.g., ASD). The loss of dendritic spines directly correlates with the loss of synaptic function. In AD, early synaptic degeneration could ultimately cause neuronal degeneration through erroneous converting signals derived from structural synaptic changes into the program of cell cycle activation [146].

The extrapolation of data from the literature, in terms of exposure levels, to study molecular mechanisms in cellular and animal models remains complex because the metal concentrations used are often high, although most of the recent data tend to take into account low exposure levels representing environmental exposures. Many questions remain to be solved, such as what are the threshold exposure concentrations showing neurotoxic effects which will depend on each element, its speciation, route and duration of exposure, the combined effects between neurotoxic contaminants, and especially according to the age of the exposed individuals, the periods of neurodevelopment being sensitive to lower metal concentrations than in adults.

A better understanding of the molecular mechanisms involved in cognitive dysfunctions associated with exposure to environmental metals will allow for devising novel prevention strategies, e.g., using supplementation with essential metals (Cu, Zn), when metallic exposure is difficult to prevent. In this respect, the restoration of balanced Cu and Zn homeostasis would be determinant to protect neurons. Organic Cu complexes have been proposed for the therapeutic redistribution of Cu in AD [147], and Zn therapy for the treatment of early AD [148]. Zinc supplementation could also restore neuronal functions in ASD [120,121].

In conclusion, there is robust evidence for toxic effects of metals towards neurotransmitter receptors, synaptic cytoskeleton, and scaffolding proteins suggesting that synapses could be the early targets of environmental metals toxicity. Nevertheless, the precise description of the molecular mechanisms of synaptic toxicity still remains largely unknown and their elucidation will allow the designing of adapted prevention strategies to treat or slow down the progression of devastating neurological diseases such as ASD and AD.
